# Whiplash injuries associated with experienced pain and disability can be visualized with [^11^C]-D-deprenyl positron emission tomography and computed tomography

**DOI:** 10.1097/j.pain.0000000000002381

**Published:** 2021-07-02

**Authors:** Mikko Aarnio, Mats Fredrikson, Erik Lampa, Jens Sörensen, Torsten Gordh, Clas Linnman

**Affiliations:** a Department of Surgical Sciences, Anesthesiology and Intensive Care Medicine, Uppsala University, Sweden; b Department of Psychology, Uppsala University, Sweden; c Department of Clinical Neuroscience, Karolinska Institutet, Stockholm, Sweden; d UCR, Uppsala Clinical Research Center, Uppsala University, Uppsala, Sweden; e PET Centre, Department of Medical Imaging, Uppsala University Hospital, Sweden; f Section of Nuclear Medicine and PET, Department of Surgical Sciences, Uppsala University, Sweden; g Department of Physical Medicine and Rehabilitation, Spaulding Rehabilitation Hospital, Harvard Medical School, Boston, United States

**Keywords:** Whiplash, Deprenyl, Inflammation, Pain, PET

## Abstract

Knowledge of etiological mechanisms underlying whiplash-associated disorders is incomplete. Localisation and quantification of peripheral musculoskeletal injury and inflammation in whiplash-associated disorders would facilitate diagnosis, strengthen patients' subjective pain reports, and aid clinical decisions, all of which could lead to improved treatment. In this longitudinal observational study, we evaluated combined [^11^C]-D-deprenyl positron emission tomography and computed tomography after acute whiplash injury and at 6-month follow-up. Sixteen adult patients (mean age 33 years) with whiplash injury grade II were recruited at the emergency department. [^11^C]-D-deprenyl positron emission tomography and computed tomography, subjective pain levels, self-rated neck disability, and active cervical range of motion were recorded within 7 days after injury and again at 6-month follow-up. Imaging results showed possible tissue injuries after acute whiplash with an altered [^11^C]-D-deprenyl uptake in the cervical bone structures and facet joints, associated with subjective pain locale and levels, as well as self-rated disability. At follow-up, some patients had recovered and some showed persistent symptoms and reductions in [^11^C]-D-deprenyl uptake correlated to reductions in pain levels. These findings help identify affected peripheral structures in whiplash injury and strengthen the idea that positron emission tomography and computed tomography detectable organic lesions in peripheral tissue are relevant for the development of persistent pain and disability in whiplash injury.

## Abstract 2

Supplemental Digital Content is Available in the Text. [^11^C]-D-deprenyl positron emission tomography and computed tomography, collected in 16 persons, with whiplash injury within 7 days of injury and at 6-month follow-up, and in 8 healthy controls, indicate that injury and related pain can be visualized and tracked.

## 1. Introduction

Whiplash-associated disorder (WAD) is a globally important clinical, social, and financial problem.^9^ Although the symptoms of WAD are well known, underlying mechanisms and the etiology of this disorder remain elusive. No peripheral tissue damage can convincingly be detected with current imaging technologies.^2,42^ The development of chronic symptoms after whiplash injuries may also be influenced by psychological and social factors^26^ as well as with changes in the central nervous system (CNS).^35,36^ This possibility has led to a discussion of whether tissue damage is even needed to initiate or maintain a WAD.^51^ Difficulties to detect and diagnose biological contributors (lesions), together with the lack of an accepted concept for what causes the symptoms in WAD, represent considerable personal, societal, and economic problems. Therefore, an objective visualization and quantification of peripheral musculoskeletal injury and possible painful inflammation in WADs would facilitate diagnosis, strengthen patients' subjective report of pain, and assist clinical decisions. In addition, it may help define new treatment targets and methods.

Positron emission tomography (PET) is an imaging tool that captures molecular and cellular biological processes and not mere anatomy or structure.^40^ Positron emission tomography technology has been used to visualize and detect inflammation in numerous clinical settings.^5,52,60^ Furthermore, whiplash injury is associated with elevated production of inflammatory mediators.^27,50^ In this context, [^11^C]-D-deprenyl PET has emerged as a novel approach to target local inflammatory processes in the musculoskeletal system and chronic WAD.^1,13,34^ As in ankle sprains, expected injuries in whiplash involve muscles, periosteal tissues, ligaments, and joints. In a recent study exploring patients with unilateral ankle sprains, we demonstrated that the molecular aspects of tissue lesions in patients presenting with inflammation and pain could be visualized, quantified, and followed over time using [^11^C]-D-deprenyl PET/computed tomography (CT).^1^ We have previously reported that mitochondrial monoamine oxidase (MAO) enzymes are a major candidate for the binding target of D-deprenyl^32^ and that D-deprenyl discriminates between low and high grades of inflammation.^31^ Therefore, [^11^C]-D-deprenyl may be valuable in the visualization and quantification of inflammation, as well as possible pain-associated processes in the peripheral tissue of WAD.

We conducted a prospective observational PET study in adult patients with acute whiplash injury and healthy controls using [^11^C]-D-deprenyl PET/CT. The aim was to characterize acute alterations and the predictive ability of [^11^C]-D-deprenyl uptake for whiplash signs and symptoms at a 6-month follow-up. We hypothesized that [^11^C]-D-deprenyl uptake would be acutely elevated, localized to anatomically relevant injured tissues, colocalized to tender points, and correlated with subjective pain experience. In addition, we hypothesized that patients with persistent pain would have prolonged elevated [^11^C]-D-deprenyl uptake at the injury sites at the 6-month follow-up.

## 2. Material and methods

### 2.1. Ethical approval

The study was approved by the Ethics Committee of the Faculty of Medicine at Uppsala University, Sweden, and by the Radiation Ethics and Safety Committee of Uppsala University Hospital, Uppsala, Sweden. All procedures were in accordance with the ethical standards of the institutional research committee and with the 1964 Declaration of Helsinki and its later amendments or comparable ethical standards. Informed consent was obtained from all participants.

### 2.2. Patients

Sixteen nonsmoking adult patients (8 males) with a whiplash injury grade II after a car crash were recruited from the emergency department at Uppsala University Hospital, Uppsala, Sweden. The mean age (±SD) of the patients was 33 years (±9). Grade II WAD refers to neck complaints accompanied by a decreased range of motion, point tenderness, or both (musculoskeletal signs).^49^ There were no explicit minimum pain levels necessary to be included, although acute pain is part of the WAD grade II criteria. No patients had a history of medical or psychiatric disease. Eight controls (4 men and 4 women, mean age 32 years (±14)) from a previous study were also included.^34^ The controls were healthy, pain free, and without current or previous neck pain.

### 2.3. Study design

The study design consisted of two [^11^C]-D-deprenyl PET/CT studies on the neck region of each patient. The first imaging session was scheduled acutely within 4 to 7 days (mean 4.9 [±1.4] days) from the injury, and all follow-up imaging sessions were scheduled 6 months (mean 221 [±24] days) after the injury. Before the examination sessions, all patients refrained from alcohol and caffeine for 12 hours and from analgesics for at least 24 hours.

### 2.4. Pain assessment and subjective ratings

Immediately before each examination, the locations of maximum tenderness were palpated and marked on an anatomical image of the neck. The patients rated their current subjective pain levels on a Numerical Pain Rating Scale (NRS) ranging from 0 (no pain) to 10 (worst imaginable pain). At the same time, a cervical range of motion (CROM) goniometer (Performance Attainment Associates, Roseville, MN) was used to measure the 6 active movements of the neck: extension, flexion, left and right lateral rotation, and left and right bending.^61^ After the imaging sessions, patients completed a whiplash questionnaire with crash description and the Neck Disability Index (NDI) questionnaire.^54^

### 2.5. Positron emission tomography and computed tomography scanning

The radioligand [^11^C]-D-deprenyl was produced at the chemistry section of the Uppsala PET Center according to a standard manufacturing procedure with previously published methods.^18,39^ All patients were investigated with a GE Discovery ST PET/CT scanner (General Electric Medical Systems, Milwaukee, WI). The scanner enables the acquisition of 47 contiguous planes of data with a distance of 3.27 mm (transaxial resolution) and 3.125 mm (axial resolution), resulting in a total axial field of view of 15.7 cm. The patients were scanned in the supine position with their head fixed and positioned in the scanner field of view originating 2 cm above the orbitomeatal line to allow data acquisition from the base of the skull down to the shoulder region.

The PET/CT investigation was initiated with a short CT scan (140 kV; auto mA 10-80 mA) for attenuation correction of the PET emission data. Each patient received an intravenous bolus of approximately 5 MBq/kg [^11^C]-D-deprenyl in the arm. Simultaneously, a dynamic emission scan (3D mode) was initiated with a predetermined set of measurements (frames of 4 × 30, 3 × 60, 2 × 300, and 3 × 600 seconds) for up to 45 minutes. Then a single 15-minute static image was collected over the lower neck area. Positron emission tomography data were reconstructed with the ordered subset expectation maximization algorithm (2 iterations and 21 subsets). A 2.57 mm wide postprocessing filter was applied to the images. Positron emission tomography data were corrected for decay, photon attenuation, scatter, random coincidences, and dead time.

### 2.6. Positron emission tomography quantification

Time activity data representing the dynamic sequence of radioactivity levels for the region of interest (ROI) in each PET/CT scan from 0 to 45 minutes were generated. The data were standardized for the administered dose of radioactivity and the patient's body weight to yield a standardized uptake value (SUV). For the SUV calculations and image analysis, the last 2 frames (25-45 minutes posttracer administration) of the dynamic data and the static data (45-60 minutes posttracer administration) were used to minimize the effect of blood flow. SUV_MAX_ was defined as the maximum value observed in a single ROI. SUV_RATIO_ was defined for each region as the ratio of SUV_MAX_ between the lesion and a cerebellar reference region.

### 2.7. Positron emission tomography and computed tomography image analysis

The image analysis was performed with Voyager (version 4.0.7; GE healthcare 2012). First, the CT image was used to delineate the anatomical ROIs in all images of each patient and control. Second, the lesions were identified visually from the PET/CT image with elevated [^11^C]-D-deprenyl uptake above the background signal, and ROIs containing the lesion were delineated manually in multiple adjacent slices. [^11^C]-D-deprenyl uptake in salivary glands and the CNS was regarded as a normal finding based on previous studies of healthy subjects. The specific lesion areas were also delineated in all images of each patient and in controls in the overlapping CT images. Finally, data from the controls were used to calculate the corresponding SUV_MAX_ and SUV_RATIO_ as well as reference intervals (mean ±2 SD) for each ROI. Anatomical and lesion ROIs demonstrating an uptake >2 SDs in any of the patients (compared with the healthy controls) were considered in the statistical analysis. These anatomical regions were identified with the help of an experienced PET radiologist (J.S.). The defined anatomical regions are approximate and can contain adjacent structures because of the physical limits of spatial resolution in PET/CT.

### 2.8. Statistical analysis

The 1-tailed nonparametric Wilcoxon signed-rank test was used to compare differences between the SUV_RATIO_ values and NRS, CROM, and NDI scores between the acute and follow-up examination. Correlations and pairwise associations between SUV_RATIO_ values and NRS, CROM, and NDI scores, and changes in these values from acute to follow-up, were assessed by Spearman rank correlation. Relationships between regional [^11^C]-D-deprenyl uptake and NRS, CROM, and NDI were also explored, see supplemental methods and results (available at http://links.lww.com/PAIN/B423).

## 3. Results

### 3.1. Patient characteristics and clinical results

Baseline characteristics of the patients and outcome measures are summarized in Table 1. NRS decreased from a median of 4.5 (interquartile range [IQR] 3.5-7) at acute investigation to 3 (0-5) at follow-up (*P* = 0.0008). During the follow-up, median self-rated disability (NDI) changed from 23 (20.5-28) to 17 (12.5-25.5) (*P* = 0.0007) and the affected neck movements (CROM) diminished from 1.5 (1-4.5) to 1 (0-2) (*P* = 0.008). A conventional CT scan of the neck, performed acutely and at follow-up on all patients, did not reveal any pathological findings.

**Table 1:** Patient characteristics and outcomes.

Case	Sex	Age	BMI	Day*		NPS			NDI			CROM†			Abnormal uptake‡					
				1st	2nd	1st	2nd	Trend	1st	2nd	Trend	1st	2nd	Trend	SUVmax			SUVratio		
															1st	2nd	Trend	1st	2nd	Trend
1	M	39	25.9	4	267	2	0	↓	20	12	↘	3	0	↓	2	12	↗	2	1	↘
2	F	20	20.1	4	229	5	0	↓	22	17	↘	1	0	↓	7	4	↘	10	5	↘
3	F	41	29.2	4	280	7	6	↘	39	31	↘	1	0	↓	11	9	↘	15	12	↘
4	M	20	20.9	6	209	8	3	↘	22	22	→	5	1	↘	2	0	↓	6	0	↓
5	M	27	23.6	6	202	2	0	↓	17	11	↘	1	1	→	0	0	→	5	0	↓
6	F	22	—	6	196	4	0	↓	26	17	↘	1	1	→	6	9	↗	10	8	↘
7	M	29	21.7	5	241	6	0	↓	15	11	↘	2	0	↓	0	0	→	0	0	→
8	F	33	—	8	227	8	6	↘	32	27	↘	6	4	↘	4	1	↘	5	1	↘
9	M	39	23.0	2	238	3	3	→	23	13	↘	0	0	→	0	0	→	1	0	↓
10	F	38	29.8	3	219	4	4	→	21	20	↘	0	0	→	1	1	→	4	6	↗
11	M	23	19.8	5	220	4	5	↗	29	15	↘	1	1	→	0	6	↑	4	8	↗
12	M	31	24.6	5	195	7	5	↘	27	28	↗	6	4	↘	0	0	→	1	2	↗
13	F	42	31.2	6	198	3	0	↓	23	12	↘	1	0	↓	1	0	↓	4	1	↘
14	F	26	24.4	6	202	8	7	↘	46	35	↘	5	2	↘	3	2	↘	9	8	↘
15	F	51	31.6	4	207	4	0	↓	19	14	↘	4	2	↘	1	0	↓	5	3	↘
16	M	45	28.1	5	216	5	3	↘	24	24	→	4	6	↗	0	0	→	0	2	↑
Average		33	25	4.9	222	4.5	3	↘	25	19	↘	2.6	1.4	↘	2.4	2.75	↗	5.1	3.563	↘
Pre–post significance§						*P* = 0.0008			*P* = 0.0007			*P* = 0.008			ns			*P* = 0.03		

* Days from whiplash injury, where first refers to acute and second to follow-up investigations.

† Number of reduced neck movements of 6 possible.

‡ Number of anatomical regions ( of 19 possible) with an abnormal D-deprenyl uptake.

§ One-tailed nonparametric Wilcoxon signed-rank test.

CROM, cervical range of motion; F, female; M, male; NPS, Numerical Pain Scale; NDI, Neck Disability Index; SUV, standardized uptake value.

### 3.2. Positron emission tomography and computed tomography images and positron emission tomography data

**Figure 1.**
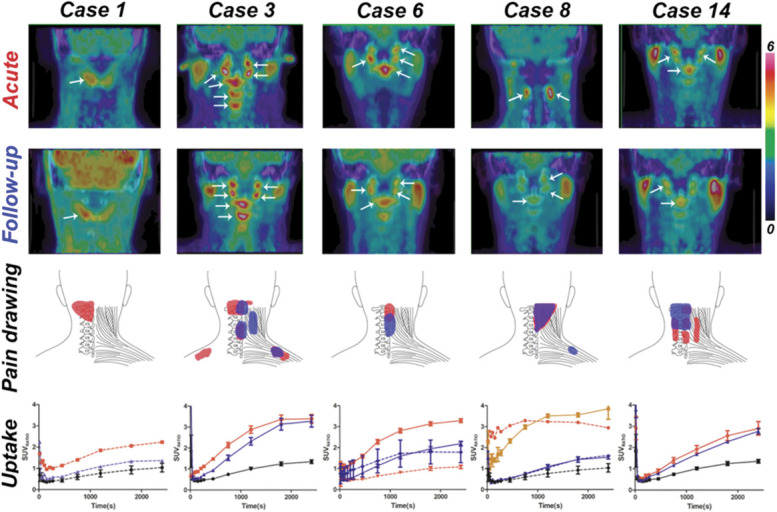
[^11^C]-D-deprenyl PET results in 5 representative cases. The 2 top rows (*Acute and Follow-up*) represent [^11^C]-D-deprenyl PET images of 5 patients with whiplash at 2 imaging sessions. The color bar indicates SUV_MAX_ values from 0 (dark blue) to 6 (red). These regions were colocalized to tenderness/pain locations (*Pain drawing*). The bottom row (*Uptake*) displays respective time activity curves (SUV_RATIO_ mean ± SE). Dashed lines represent muscles and solid lines bone structures, joints, or both. Red color indicates acute investigation, blue color follow-up, and black color corresponding anatomical regions in control patients. Case 1 represents increased [^11^C]-D-deprenyl uptake in a muscle (m. obliquus inferior, pointed by a white arrow) that was clearly reduced at follow-up. Case 3 represents increased uptake in multiple regions (arrows that point to the occipital condyles, upper facet joints, and vertebral bodies C2-C4) on both imaging occasions. Case 6 represents increased uptake in upper facet joints, occipital condyles, and the vertebral body of C2 (arrows) that was reduced at follow-up. An increase in the intensity of uptake in a muscle (m. rectus capitis posterior major) was seen at the follow-up scan. Case 8 represents increased uptake in a muscle (m. interspinal C2-C3, arrows at the acute session) and in bone structures (arrows at the follow-up session) with an equal decrease in the intensity of uptake at the follow-up scan. Case 14 represents increased uptake in the occipital condyles and the vertebral body of C2 (arrows) on both imaging occasions. Please note that parotid gland uptake, visually evident bilaterally in case 3, 6, 8, and 9, was considered normal because this was also observed in healthy controls. PET, positron emission tomography.

Increased [^11^C]-D-deprenyl uptake was visually evident and quantitatively observed in multiple locations in upper neck regions in the patients as compared with the healthy controls (Fig. 1). The lower neck regions did not show significant [^11^C]-D-deprenyl uptake. Nineteen anatomical regions showed an increased [^11^C]-D-deprenyl uptake with SUV_MAX_ or SUV_RATIO_ >2 SD above the mean uptake in the corresponding anatomical regions of the healthy controls (Table 1; and Supplemental Fig. 1, available at http://links.lww.com/PAIN/B423). A SUV_MAX_ >2 SD above the mean in the healthy controls was observed in 14/32 (44%) of the patients' scans with a median SUV_MAX_ of 5.3 (IQR 4.4-6.0) acutely and 4.5 (IQR 3.8-6.1) at follow-up. SUV_RATIOS_ >2 SD of the mean of the healthy controls were observed in 22/32 (81%) of the patients' scans with a median SUV_RATIO_ of 2.0 (IQR 1.7-3.0) at acute scans and 2.0 (IQR 1.7-2.4) at follow-up. During the acute investigations, abnormal [^11^C]-D-deprenyl uptake (SUV_RATIO_) was observed in the muscle (10 of 16 patients or 63%), bone structure (12/16 or 75%), facet joint (10/16 or 63%), occipital condyle (5/16 or 31%), groove for spinal nerve (4/16 or 25%), and temporomandibular joint (3/16 or 19%) in patients. At follow-up, abnormal [^11^C]-D-deprenyl uptake (SUV_RATIO_) was observed in the muscle (6/16 or 38%), bone structure (6/16 or 38%), facet joint (7/16 or 44%), occipital condyle (5/16 or 31%), groove for spinal nerve (2/16 or 13%), and temporomandibular joint (2/16 or 13%) in patients. The areas of elevated uptake in the upper neck were colocalized to painful locations and maximum tenderness points (Fig. 1). The dynamics of the PET evaluations was characterized by a rapid initial increase in the first few frames, followed by a more gradual increase and then generally plateauing in the last frames (Fig. 1).

The number of anatomical regions with an abnormal uptake (SUV_RATIO_) was significantly and positively associated with the NRS ratings (Spearman rank correlation coefficient 0.45, *P* = 0.009) and the NDI (Spearman rank correlation coefficient 0.39, *P* = 0.028).

**Figure 2.**
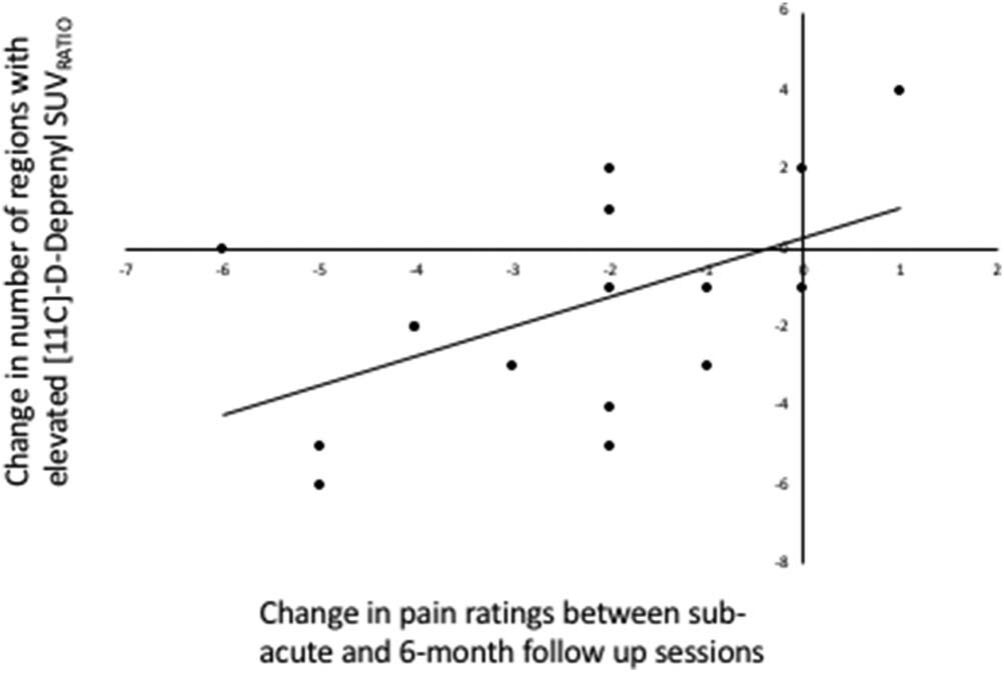
Change in NRS pain ratings and change in the number of regions with elevated [^11^C]-D-deprenyl SUV_RATIO_. Subjects pain diminished over time in most participants (*P* = 0.002), and reductions were correlated to changes in the [^11^C]-D-deprenyl SUV_RATIO_, Spearman rank correlation coefficient 0.5 (*P* = 0.048). NRS, Numerical Pain Rating Scale; SUV, standardized uptake value.

At follow-up, the number of regions with elevated uptake (SUV_RATIO_) was significantly fewer across subjects (*P* = 0.017), but still more prevalent than in the healthy control group (*P* < 0.001). Changes (typically reductions) in NRS ratings between the acute stage and follow-up were significantly correlated to changes (typically reductions) in the number of regions with an abnormal SUV_RATIO_ uptake (Spearman rank correlation coefficient 0.5, *P* = 0.048), Figure 2.

Relationships between specific anatomical locations and NDI, NRS, and CROM are reported in supplemental materials (available at http://links.lww.com/PAIN/B423).

## 4. Discussion

Molecular aspects of inflammation and tissue injuries after whiplash can be visualized, objectively quantified, and followed over time with [^11^C]-D-deprenyl PET/CT. We found an association between imaging findings in the upper cervical bone structures and patient self-report of pain and disability. At 6 months, patients had significantly improved neck disability ratings, CROM, pain levels, and significantly fewer regions with elevated uptake. Reductions in pain and number of regions with elevated tracer uptake were also linearly correlated. Nonetheless, the patients still experienced pain, disability, and reduced range of motion, and elevated [^11^C]-D-deprenyl uptake was still evident. This suggests that healing was progressing, but still ongoing at 6-month follow-up in most patients, and that [^11^C]-D-deprenyl PET holds potential to capture this process.

### 4.1. Localization of elevated [^11^C]-D-deprenyl uptake

Our study demonstrates an elevated uptake of [^11^C]-D-deprenyl in several anatomical regions. This heterogeneous injury panorama can be explained by biomechanical studies of whiplash injury.^8,12^ Tracer uptake in the upper cervical regions was expected because (1) we observed them in our previous study on chronic WAD^34^ and (2) these structures are involved in stabilizing the head-neck complex and most of the extension, flexion and rotation of the neck occurs between the occiput, C1 and C2.^3^ Many of these previously described candidate lesions have been identified in animal studies, postmortem observations, and cadaveric models but have not been identified with current imaging modalities in patients with WAD.

The [^11^C]-D-deprenyl uptake was mostly observed in bone structures and especially in upper vertebral bodies and facet joints (together with surrounding soft tissues). There is growing evidence that facet joints and capsules can be injured in whiplash and that these can be the source of pain.^58^ The clinical relevance of other tissue injuries is less clear, but cadaveric, postmortem, and animal whiplash injury models have systematically demonstrated injuries in the upper cervical discs and outer rim of the vertebral bodies and endplates. Endplate and adjacent bone marrow changes have been observed in patients with whiplash.^29^ These traumatically induced changes reflect hypervascularity because of inflammation (Modic type 1) and fatty replacements of the red bone marrow (Modic type 2).^28,43^

The observed [^11^C]-D-deprenyl uptake and possible injury in the region of occipital condyles (atlanto-occipital joints) indicate another, less established potential injury mechanism. Widening of the atlanto-occipital joint spaces, which indicates possible ligamentous injury followed by instability, has been described.^33,44^ We further observed an elevated [^11^C]-D-deprenyl uptake in the extensor muscles of the neck in most scans. Structural changes in the muscles may play a role in the development of WAD,^14,16^ and muscle tears, hematomas, strains, and perimuscular fluid have been visualized in MRI in patients with whiplash.^2^ A fatty infiltration, especially in suboccipital and upper segmental extensor muscles (rectus capitis posterior minor/major and interspinales muscles), has been previously shown.^15^ In our study, tracer uptake in the temporomandibular joint was present in 3 patients. This uptake was already evident at the acute investigation, which may support the hypothesis of direct trauma to the joint (called mandibular whiplash injury) and that the whiplash trauma may be an aggravating factor for the progression of temporomandibular disorders.^20,48^ Unfortunately, MRIs were not obtained in the present cohort, so we cannot directly compare the sensitivity and specificity between MRI and [^11^C]-D-deprenyl.

### 4.2. Pain, disability, and [^11^C]-D-deprenyl uptake

Contrary to the idea that whiplash only affects soft tissues, our study shows a positive association between [^11^C]-D-deprenyl uptake in the injured bone and joint structures and self-reported pain, self-rated disability, and pain localization. The colocalization between tracer uptake and pain locale was visually evident in several cases. However, a direct relation between tissue injury and self-report of pain may be an oversimplification: Nociceptive signal transmission from a tissue injury is under strong peripheral modulation but also from the CNS (spinal cord, brain stem, and forebrain).^11,53^ Furthermore, social, economic, psychological, and genetic factors influence all pain experiences, including WAD.^23,26^ Still, our findings support the hypothesis that the experienced pain and disability in whiplash may be driven by an ongoing peripheral nociceptive source. The present findings also support studies suggesting inflammatory processes are involved in the development and presentation of chronic WAD.^27,34,50^

Initial pain intensity and neck-related disability are the most consistent prognostic factors in patients with acute WAD.^6,26,56,57^ By contrast, the best predictor of outcome is time, that is, the prognosis for whiplash injuries will be worse with a longer duration of symptoms.^19^ In this study, the number of anatomical regions with an elevated [^11^C]-D-deprenyl uptake shows an association with NRS and NDI. This finding supports the notion that [^11^C]-D-deprenyl uptake is related to the extent and severity of the injury.^10^ Of note, the correlations between pain ratings and [^11^C]-D-deprenyl uptake were modest. This is expected, as multiple factors other than tissue damage will influence individual pain ratings,^45^ including the anchoring of the rating scale.^55^

Upper facet joints and vertebral bodies show the strongest association between pain and [^11^C]-D-deprenyl uptake (supplemental analysis, available at http://links.lww.com/PAIN/B423). The strongest evidence concerns pain from the facet joints, although the facet joint injury in patients with WAD has not previously been visualized. Facet joint capsules as the “source” of pain in WADs has been validated in animal models^30,58,59^; in human treatment studies, diagnostic blocks and radiofrequency neurotomy can abolish neck pain from these joints.^4,37,38,46^ Chronic pain can be traced to facet joints in about 50% of patients with whiplash in these studies, which can be compared with [^11^C]-D-deprenyl uptake in 44% of the patients at follow-up in our study. Particularly, noteworthy is that local inflammation and not pure mechanical injury or development of osteoarthritis has been linked to generation of pain from facet joints.^24^ Endplate and bone marrow changes (Modic changes) have been associated with low back pain,^28^ and the Modic changes in neck pain patients have been described as a dynamic phenomenon without completely disappearing during follow-up.^41^ We did not observe an association between pain and possible muscle injury but see supplementary materials (available at http://links.lww.com/PAIN/B423) for further details on muscle and dorsal root ganglia observations.

We saw indications of associations between initial [^11^C]-D-deprenyl uptake in the upper bone structures and joints and later pain and disability. A possible explanation could be a more severe initial injury or a localization in which the healing process is abnormal. New regions of tracer uptake at follow-up might be explained by ligament insufficiency and impaired cervical position sense (proprioception) that are common in cervical pain.^22,47^ These changes can alter the kinematics of the neck with altered shear load and posture and initiate novel inflammatory processes. Such adaptation-related increases in [^11^C]-D-deprenyl uptake have previously been observed in both feet after recovery from a one-sided ankle sprain.^1^ Consequently, new uptake regions could be the first signs of adaptive or degenerative changes in the neck tissues. Of note, MRI examination can reveal pronounced degenerative changes in asymptomatic patients,^2,7,17,21^ but these changes are less frequent in patients aged <40 years.

### 4.3. The limitations of the study and [11C]-D-deprenyl uptake mechanism

The main limitations of the study are the limited sample size, and the exact uptake mechanism of D-deprenyl in musculoskeletal injury remains elusive. D-deprenyl is a weak lipophilic base, and a possible local increase in blood flow in neck tissues needs to be considered when interpreting [^11^C]-D-deprenyl uptake. To reduce the effects of blood flow, a semiquantitative measurement of the radioactivity concentration in tissue with a reference region (SUV_RATIO_) was used. This approach minimizes (but may not eliminate) the blood flow component. A full tracer kinetic modeling with arterial blood sampling to measure radioactivity and D-deprenyl metabolites would be needed to exclude blood flow effects. According to the available data, the main binding target for D-deprenyl is MAO enzymes in the cells that are engaged in processes involved in inflammation,^31,32^ although other protein targets distinct from MAO-B, including sigma-1,^25^ cannot be excluded. The complex role of MAO enzymes in peripheral inflammation warrants further studies.

The small sample size, typical for PET studies, was limited because of the high costs of PET and feasibility concerns. We conclude that [^11^C]-D-deprenyl uptake is associated with pain and disability at 2 time points. Participants exposed to motor-vehicle impacts of similar forces but without experiencing neck pain would be an ideal control group to account for the specificity of neck symptoms and D-deprenyl uptake and also the psychological stress of a traumatic vehicle crash. Prospective cohort studies are needed to elucidate the predictive and diagnostic value of PET/CT in whiplash injury.

## 5. Conclusion

This study provides further evidence that tissue injury and inflammation in whiplash injury can be objectively visualized and quantified using PET/CT, suggesting lesions in peripheral tissue are relevant for the development of persistent pain and disability in whiplash injury. Recognition of these affected structures should advance general knowledge of whiplash disorders and facilitate individualized treatment interventions.

## Conflict of interest statement

The authors have no conflicts of interest to declare.

## Appendix A. Supplemental digital content

Supplemental digital content associated with this article can be found online at http://links.lww.com/PAIN/B423.

## Supplementary Material

SUPPLEMENTARY MATERIAL
