# Supplementary material for: Whiplash injuries associated with experienced pain and disability can be visualized with [11C]-D-deprenyl positron emission tomography and computed tomography
Source: Pain. 2021 Jul 2;163(3):489–95. doi: 10.1097/j.pain.0000000000002381 (PMC8832543; doi:10.1097/j.pain.0000000000002381)
Supplement: SUPPLEMENTARY MATERIAL [file jop-163-0489-s001.pdf]

Supplemental material

To explore anatomical relationships to behavioral outcomes, Spearman's rank correlations were performed between regional uptake and NRS, CROM and NDI. With 57 pairwise correlations, we would expect about three significant findings by chance alone. Therefore, to adjust for multiple comparisons a multivariate rank-based test of association was performed to formally test for associations. The null distribution for the multivariate test statistic was obtained by randomly permuting the NRS, CROM and NDI values 10,000 times while keeping the SUV values fixed, thereby breaking any associations that may exist in the data. Adjustment for multiple testing was done using the step-down method (*1*) in which the dependency of the test statistics are taken into account. The step-down method is generally less conservative than the Bonferroni method commonly applied in multiple testing situations. All analyses were done using R (*2*) version 3.3.1 with the coin (*3*) package.

Pairwise associations as measured by Spearman's rank correlation between uptake at different points and outcomes (NRS, CROM and NDI) revealed multiple associations between uptake (SUV_{RATIO}) in upper bone structures and facet joints for the NDI and NRS but not for CROM (Fig. 2). The [^{11}C]-D-deprenyl uptake in muscles and soft tissues was associated with CROM, the NDI, or both at the 6-month follow-up only. The upper bone structures showed an association between acute [^{11}C]-D-deprenyl uptake and the NRS and NDI at the 6-month follow-up (Supplemental Figure 1). However, after taking the number of tests performed into account, no statistical significance remained between the [^{11}C]-D-deprenyl signal intensity and numerical values of the NRS, CROM or NDI.

Pain, and especially referred pain from muscles and tendons, is believed to be common in WAD, but MRI studies do not support the conclusion that muscle injuries contribute to persistent symptoms (*4, 5*). In our study pain after whiplash was not associated with possible tissue injury in muscles, but at follow-up the tracer uptake in neck muscles was associated with reduced neck movements and self-rated disability. Disuse, lack of range of movements and increased intramuscular adipose tissue possibly contribute to the elevated tracer uptake (*6-8*). Other lesions show no or even a negative association with reduced neck movements, in line with studies demonstrating that CROM had no significant effect on recovery (*9, 10*). In our study there were no associations between uptake in temporomandibular joints and measured outcomes, suggesting that this uptake is a separate comorbid condition to whiplash. There was also an association between pain, disability and uptake near dorsal root ganglia and spinal nerve at C4 level, which suggests that injuries near these regions may have clinical relevance, as evidenced by recent MRI magnetization transfer imaging of the cervical spinal cord in whiplash patients (*11*).

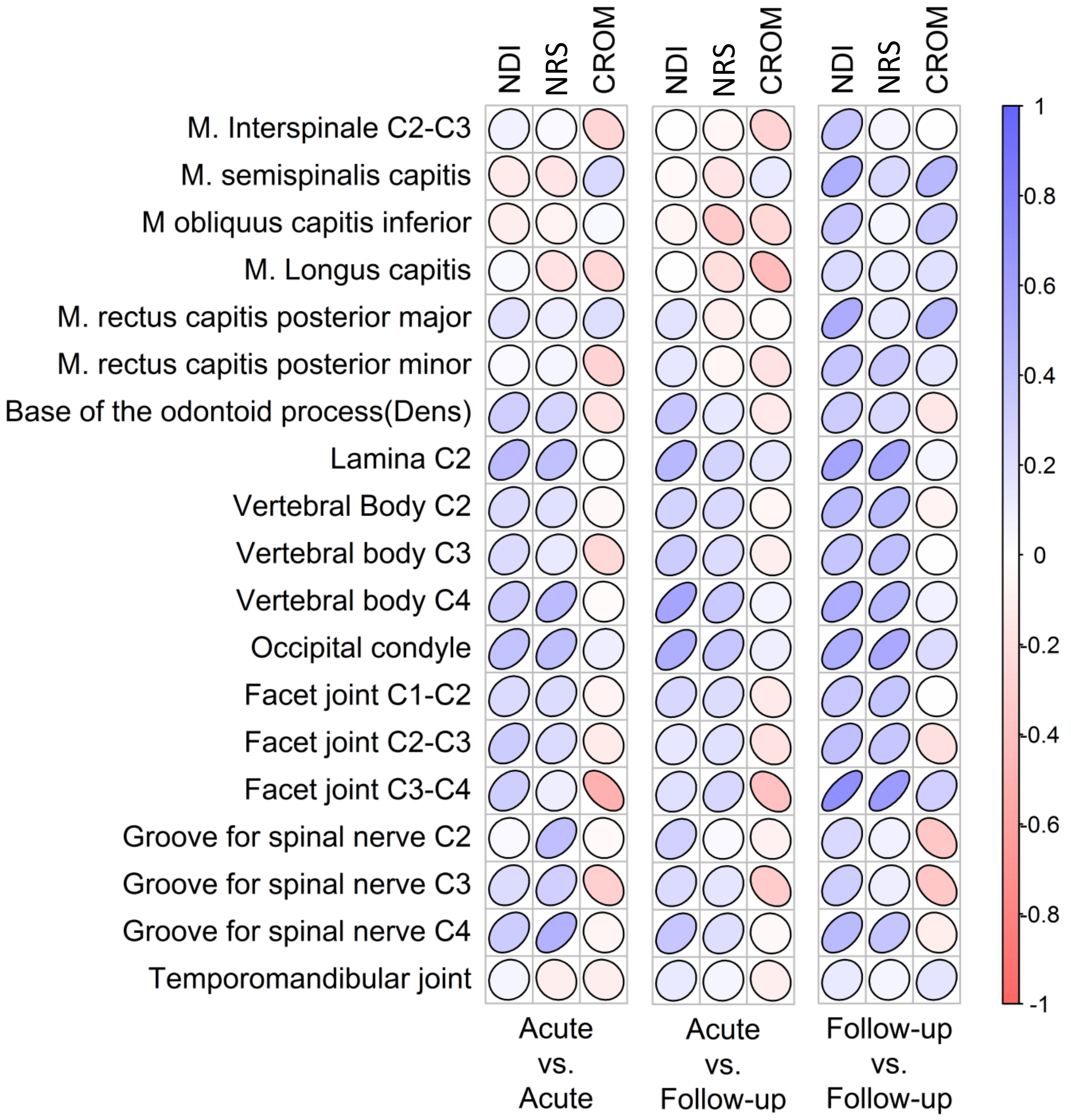

Supplemental Figure 1. Anatomical regions with uptake >2 standard deviations compared with healthy controls. Spearman's rank correlation coefficients between uptake (SUV_{RATIO}) and measured outcomes. Blue represents a positive and red a negative correlation. Color intensity and ellipse shape are proportional to the absolute coefficient size. For example, a symmetrical white circle represents a zero correlation coefficient while a tight blue ellipse tilted to the right

mirrors a positive correlation and red to the left a negative correlation. NDI - Neck Disability Index, NRS – Numeric Pain rating Scale, CROM - cervical range of motion.

Supplementary references

1. P. H. Westfall, S. S. Young. (Biometrics, 1993), vol. 49, pp. 941-945.
2. R. C. Team. (R Foundation for Statistical Computing, Vienna, Austria, 2016).
3. T. Hothorn, K. Hornik, M. A. van de Wiel, A. Zeileis. (The American Statistician, 2006), vol. 60, pp. 257-263.
4. G. E. Borchgrevink *et al.*, MR imaging and radiography of patients with cervical hyperextension-flexion injuries after car accidents. *Acta radiologica* **36**, 425-428 (1995).
5. H. R. Ronnen *et al.*, Acute whiplash injury: is there a role for MR imaging?--a prospective study of 100 patients. *Radiology* **201**, 93-96 (1996).
6. J. Elliott *et al.*, The temporal development of fatty infiltrates in the neck muscles following whiplash injury: an association with pain and posttraumatic stress. *PLoS One* **6**, e21194 (2011).
7. S. E. Larsson, M. Alund, H. Cai, P. A. Oberg, Chronic pain after soft-tissue injury of the cervical spine: trapezius muscle blood flow and electromyography at static loads and fatigue. *Pain* **57**, 173-180 (1994).
8. C. Linnman *et al.*, Elevated [11C]-D-deprenyl uptake in chronic Whiplash Associated Disorder suggests persistent musculoskeletal inflammation. *PLoS One* **6**, e19182 (2011).
9. D. M. Walton, J. Pretty, J. C. MacDermid, R. W. Teasell, Risk factors for persistent problems following whiplash injury: results of a systematic review and meta-analysis. *J Orthop Sports Phys Ther* **39**, 334-350 (2009).
10. M. Williams, E. Williamson, S. Gates, S. Lamb, M. Cooke, A systematic literature review of physical prognostic factors for the development of Late Whiplash Syndrome. *Spine (Phila Pa 1976)* **32**, E764-780 (2007).
11. M. A. Hoggarth *et al.*, Macromolecular changes in spinal cord white matter characterize whiplash outcome at 1-year post motor vehicle collision. *Scientific reports* **10**, 22221 (2020).
